# Time to initiate complementary feeding and associated factors among mothers with children aged 6–24 months in Tahtay Maichew district, northern Ethiopia

**DOI:** 10.1186/s13104-019-4061-2

**Published:** 2019-01-14

**Authors:** Ermyas Brhane Reda, Alemayehu Shimeka Teferra, Measho Gebreslassie Gebregziabher

**Affiliations:** 1 Department of Public Health, College of Health Sciences, Aksum University, P.O. Box: 298, Aksum, Ethiopia; 2 Department of Epidemiology and Biostatistics, Institute of Public Health, College of Medicine and Health Sciences, The University of Gondar, P.O. Box 196, Gondar, Ethiopia; 3 Department of Health Service Management and Health Economics, Institute of Public Health, College of Medicine and Health Sciences, The University of Gondar, P.O. Box 196, Gondar, Ethiopia

**Keywords:** Time to initiate, Complementary feeding, Children age 6–24 months

## Abstract

**Objective:**

In Ethiopia, only 51% of the infants start complementary feeding on time. Therefore this study is aimed to determine the time to initiate complementary feeding and associated factors among mothers with children aged 6–24 months in Tahtay Maichew district, northern Ethiopia. A retrospective follow up study was conducted among 639 mothers who had children aged 6–24 months. Bi-variable and multi-variable Cox regressions were conducted and statistical significance was declared at P-value < 0.05 and 95% confidence level.

**Results:**

The median age for the initiation into complementary feeding was 6.00 months. Being government employee [AHR = 1.67, 95% CI 1.10–2.53], having educated husband [AHR = 2.08, 95% CI 1.22–3.86], birth preparedness [AHR = 3.74, 95% CI 1.49–9.94], growth monitoring [AHR = 5.79, 95% CI 2.60–12.88], ability to know exact time to introduce complementary feeding [AHR = 4.93, 95% CI 1.94–12.50], and paternal support [AHR = 4.99, 95% CI 2.02–12.34] were significantly associated with the time to initiate into complementary feeding. Therefore, establishing breast feeding centres at work place and extending maternity leave for reasonable months are important to improve timely initiation into complementary feeding.

**Electronic supplementary material:**

The online version of this article (10.1186/s13104-019-4061-2) contains supplementary material, which is available to authorized users.

## Introduction

Adequate nutrition from birth to 2 years of age is a critical window for the promotion of optimal growth, health, and behavioural development [1]. For an optimal growth of an infant, the World Health Organization recommends exclusive breast feeding for the first 6 months and the introduction of complementary feeding (CF) at the age of 6 months [2].

Adherence to infant feeding recommendations is particularly important in low-income countries. In areas with poor food or water hygiene, early introduction of complementary foods is associated with increased morbidity from diarrheal diseases and the development of malnutrition [3–5]. Too long delay in introducing appropriate complementary foods may, however, lead to nutritional deficiencies of iron, zinc, calcium, sometimes vitamin A, and riboflavin because of the relatively low density of these nutrients in breast milk 6–9 months after birth [6]. Many Studies conducted in different part of the world indicated the many children did not start complementary feeding at the right time. Early introduction of CF is a common problem [7–10].

The case in Ethiopia is not different from the others. Despite, the fact that the government had been taking many measures including development of the national Infant and young child feeding (IYCF) strategy, only 51% of the infants are initiated into CF on time [11–13].

Studies suggest that many possible factors were associated with the time to initiate CF including mothers’ educational status, fathers’ occupation, fathers’ educational status, husband support, place of delivery, having ANC and PNC, sex of the child [11, 14–16], birth preparedness [14], birth order [17, 18] mothers access to newspapers, radio or television [5, 17, 19] having growth monitoring [20] and ability to know the exact time to introduce CF [5, 20–26].

Despite, numerous studies on CF practices and associated factors in many parts of the country they limited themselves to whether mothers introduced complementary feeding on time or not and failed to determine the median time for introducing complementary feeding. Therefore, the aim of this study was to determine the time to initiate complementary feeding and associated factors among mothers with aged 6–24 months in Tahtay Maichew district.

## Main text

### Methods

#### Study design and setting

A retrospective follow up study was conducted from February 15, to March 15, 2016. The study was conducted in Tahtay Maichew district which is found in northern Ethiopia 1041 kms away from Addis Ababa, the capital city of Ethiopia.

#### Study population

All mothers having children 6–24 months age were the source population.

#### Sample size and sampling technique

The sample size was calculated using the double population proportion formula with the following assumptions: 95% confidence level, 80% power, ratio of non-exposed to exposed 1, outcome variable among non exposed 20.9% and calculated using Epi Info7 [21]. The sample size was 639 after considering 10% non-response rate and 1.5 design effect. The sample was obtained using the systematic multi-stage sampling technique.

#### Study variables and their measurement

The event in this study was initiation of complementary feeding coded as one otherwise as zero. The time variable in this study was time from birth to initiation complementary feeding. Factors included in the model as an independent variables were: Socio demographic and economic factors, knowledge and practice related factors and reproductive and health service utilization related factors.

#### Operational definitions

Time to initiate CF: the time at which the mother starts giving the child either solid, semisolid or liquids other than breast milk or formula feeding.

Ability to know the time to initiate CF: a mother answers that the time to initiate CF is exactly at 06 months or 180 days after birth.

#### Data processing and analysis

Data were collected using pre-tested and interviewer-administered sets of questionnaire. The collected data were checked for completeness and consistency and then, entered into Epi Info7. The entered data were exported to STATA version 11 for analysis.

Life table was constructed to estimate the probabilities of initiation of CF over time. Kaplan–Meier survival curve together with log-rank test was fitted to test for the presence of difference in time into initiation of CF between categorical variables. Before the inclusion of predictors to the multivariable Cox regression analysis, fulfillment of the model assumption was checked using the goodness-of-fit test.

Both bi-variable and multivariable Cox proportional hazards model were used to identify factors that affected the time to initiate complementary feeding. Variables with a P-value of ≤ 0.05 in multivariable Cox regression analysis were considered as factors associated with the time to initiate into complementary feeding at a 95% confidence level.

### Results

#### Socio-demographic, reproductive health and health service utilization characteristics of mothers

Out of 639 participants included in the study only 633 participants provide a complete response with response rate of 99%. The median age of the mothers, with inter quartile range was 27 and 9 years, respectively. The lowest and highest age was ranged from 16 to 47 years. The median ages of the children were 12 months. Five hundred eighty-six (92.58%) of mothers were married and 58.77% of mothers were farmers. Five hundred seventy (90.05%), 61.77%, and 69.04% of the mothers had one or more ANC, PNC, and growth monitoring follow up, respectively (Table 1).

**Table 1:** Socio-demographic, reproductive health and health service utilization characteristics of mothers with children aged 6–24 months in Tahtay Maichew district, northern Ethiopia, 2016, [N = 633]

Variable	Category	Number	Percent
Residence	Rural	501	79.15
	Urban	132	20.85
Age of the mother (in years)	15–24	241	38.07
	25–34	284	44.87
	35–44	99	15.64
	45 and above	09	1.42
Religion	Orthodox	580	91.63
	Muslim	53	8.37
Marital status	Married	586	92.58
	Divorced	25	3.95
	Separated	17	2.69
	Widowed	5	0.79
Maternal education	No formal education	226	35.70
	Primary school (1–8)	281	44.39
	High school (9–12)	100	15.80
	Diploma and above	26	4.11
Husband’s education	No formal education	223	35.51
	Primary school (1–8)	216	34.39
	High school (9–12)	137	21.82
	Diploma and above	52	8.28
Mothers employment	Farmer	372	58.78
	House wife	185	29.23
	Private	41	6.48
	Government	35	5.53
Husbands employment	Farmer	378	61.62
	Private	129	20.54
	Government	61	9.71
	Daily laborer	51	8.12
Wealth index	Poor	211	33.33
	Medium	214	33.81
	Rich	208	32.86
Sex of index child	Male	336	53.08
	Female	297	46.92
Age of index child (in months)	6–12	360	56.87
	13–18	140	22.12
	19–23	133	21.01
ANC follow up	Yes	570	90.05
	No	63	9.95
Birth preparedness	Yes	579	91.48
	No	54	8.52
Place of delivery	Health facility	558	88.15
	Home	75	11.85
Mode of delivery	Spontaneous vaginal delivery	610	96.37
	Cesarean section	23	3.63
PNC follow up	Yes	391	61.77
	No	242	38.23
Counseling during ANC or PNC	yes	518	81.83
	No	115	18.17
Current contraceptive use	Yes	426	67.3
	No	207	32.7
Growth monitoring	Yes	437	69.04
	No	196	30.96

#### Knowledge and practice related characteristics of mothers

Three hundred eighty-two (60.35%). of the mothers knew the exact time to initiate complementary feeding. Four hundred fifty-six (72.2%) mothers started complementary feeding for their index children with cereal based foods, and 73.46% used cup and spoon for feeding.

#### Time to initiate into complementary feeding

The participants were assessed retrospectively for 3794 person-months. The overall incidence density of complementary feeding was 156.56 per 1000 persons-months. The median time for the initiation into complementary feeding was 6.00 [95% CI 5.94–6.05] months. About 30.17% [95% CI 26.56–33.75], 53.40% [95% CI 49.49–57.29], and 16.42% [95% CI 13.53–19.32] started complementary feeding early, on time, and late, respectively.

#### Factors associated with time to initiate into complementary feeding

In the multi-variable Cox regression analysis maternal employment (being government employed [AHR = 1.67]), having formally educated husband [AHR = 2.08], birth preparedness [AHR = 3.74], growth monitoring status [AHR = 5.79], ability to know the exact time to introduce into complementary feeding [AHR = 4.93], and husband support [AHR = 4.99] were significantly associated with the time to initiate CF.

Specifically, the hazard of introducing CF among government employed mothers was 1.67 times higher as compared with housewives [AHR = 1.67, 95% CI 1.10–3.53]. Such hazard was 2.09 times higher among mothers whose husbands had no formal education when compared to mothers whose husbands had primary and above education [AHR = 2.09, 95% CI 1.22–3.86]. The risk of introducing CF among mothers who did not have birth preparedness was 3.74 times higher than their counter parts [AHR = 3.74, 95% CI 1.49–9.94].

**Fig. 1:**
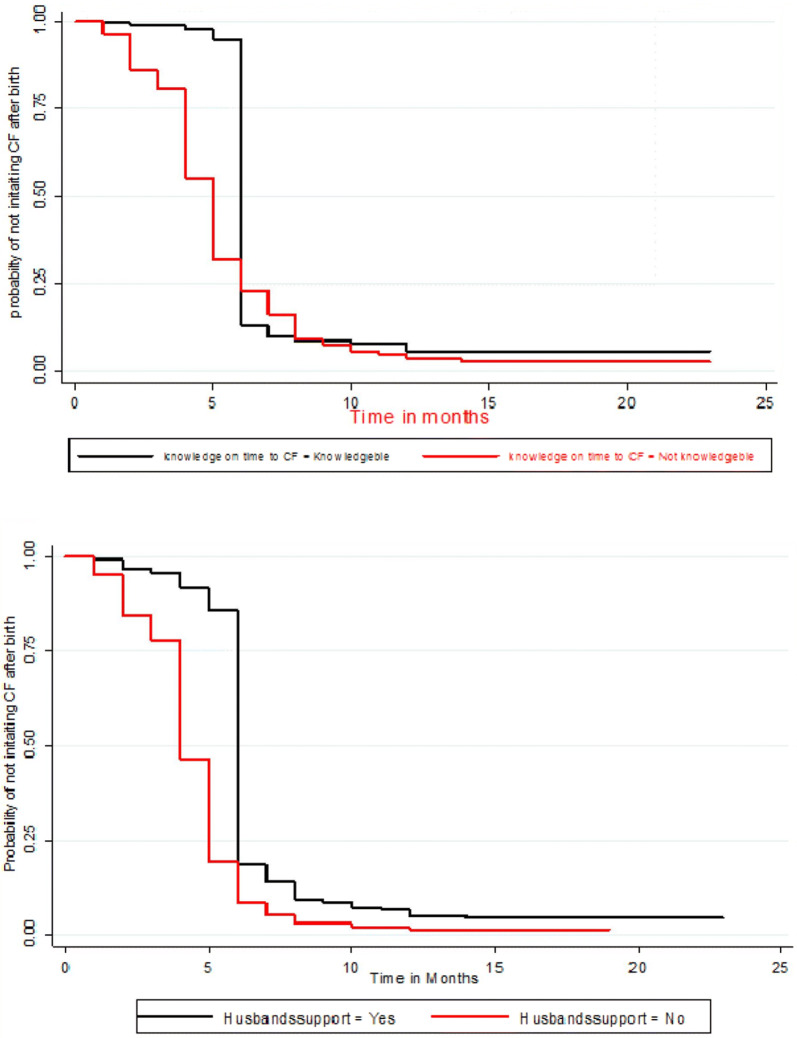
Kaplan–Meier survival plot of factors associated with time to initiate into complementary feeding among mothers with children 6–24 months in Tahtay Maichew district, North Ethiopia

The risk of introducing CF among mothers who did not take their children for growth monitoring was 5.79 times higher as compared to mothers who took their children for growth monitoring [AHR = 5.79, 95% CI 2.60–12.88]. Such risk was 4.93 times higher among mothers who did not know the exact time to introduce CF as compared to mothers who knew the exact time to introduce CF [AHR = 4.93, 95% CI 1.94–12.50]. Among mothers who did not have husband support during child feeding, the hazard was 4.99 times higher compared to mothers who had husband support on child feeding [AHR = 4.99 95% CI 2.02–12.34] (Table 2 and Fig. 1).

**Table 2:** Bi-variable and multivariable Cox regression of variable associated with the time to initiate into complementary feeding among mothers who have index children aged 6–24 months, in Tahtay Maichew district, northern Ethiopia, 2016 (N = 633)

Variable	Start CF		CHR (95% CI)	AHR (95% CI)
	Yes (%)	No (%)		
*Maternal employment*				
House wife	172 (92.97)	13 (7.03)	1	1
Government employment	34 (97.14)	1 (2.86)	1.22 (0.85–1.77)	1.67 (1.10–2.53)*
Private	34 (82.93)	7 (17.07)	0.76 (0.52–1.09)	1.255 (0.76–2.08)
Farmer	354 (95.16)	18 (4.84)	1.18 (0.98–1.42)	1.64 (0.82–3.23)
*Husbands education*				
No formal education	210 (94.17)	13 (5.83)	1.30 (1.10–1.538)	2.08 (1.22–3.86)*
Primary education and above	379 (93.58)	26 (6.42)	1	1
*Wealth index*				
Poor	195 (92.42)	16 (7.58)	1	1
Medium	199 (92.99)	15 (7.01)	1.06 (0.87–1.30)	0.99 (0.77–1.27)
Rich	200 (96.15	8 (3.85)	1.20 (0.99–1.46)	1.05 (0.79–1.40)
*ANC follow up*				
Yes	537 (94.21)	33 (5.79)	1	1
No	57 (90.48)	6 (9.52)	2.15 (1.63–2.84)	1.58 (0.60–4.21)
*Birth preparedness*				
Yes	541 (93.92)	35 (6.06)	1	1
No	53 (92.98)	4 (7.02)	2.20 (1.58–2.80)	3.74 (1.49–9.39)*
*PNC follow up*				
Yes	365 (93.35)	26 (6.65)	1	1
No	229 (94.63	13 (5.37)	1.49 (1.27–1.76)	1.15 (0.94–1.41)
*Getting counseling at ANC/PNC*				
Yes	484 (93.44)	34 (6.56)	1	1
No	110 (95.65)	5 (4.35)	1.87 (1.51–2.30)	0.82 (0.58–1.17)
*Contraceptive use*				
Yes	401 (94.13)	25 (5.87)	1	1
No	193 (93.24)	14 (6.76)	1.23 (1.03–1.46)	0.86 (0.70–1.05)
*Growth monitoring*				
Yes	410 (94.04)	26 (5.96)	1	
No	184 (93.40)	13 (6.60)	1.55 (1.46–2.07)	5.79 (2.60–12.88)**
*Ever heard about time to start CF before*				
Yes	478 (93.00)	36 (7.00)	1	1
No	116 (97.48)	3 (2.52)	1.99 (1.62–2.45)	1.13 (0.82–1.56)
*Ability to know the exact time to start CF*				
Yes	354 (92.67)	28 (7.33)	1	1
No	240 (93.84)	11 (6.16)	1.96 (1.65–2.32)	4.93 (1.94–12.50)*
*Husband support*				
Yes	443 (93.46)	31 (6.54)	1	1
No	151 (98.05)	3 (1.95)	2.79 (2.31–3.38)	4.99 (2.02–12.34)**

* P value < 0.05, ** P value < 0.001, *1* reference group

### Discussion

This study showed that the median age to initiate into CF was 6.00 months. Being government employee, husbands’ educational status, birth preparedness, growth monitoring, ability to know the exact time to introduce complementary feeding, and husband support were factors significantly associated with the time to initiate into CF.

The median age in this study is in line the study in Mekele town (6.00 months) and World Health Organization recommendation [2, 14], but it is slightly higher compared to the findings of studies in China and the United Kingdom [5, 11]. Compared to the findings of studies in Malawi and Nairobi ours was found to be higher [12, 13]. The extended timing to initiate into CF in the study area can be explained by the expansion of the Health Extension Program and the deployment of health development army in the study area.

Government employed mothers were 1.67 times at higher hazard of introducing CF as compared to that of housewives. Supportive findings were reported from China [11], West African countries [20], Kamba district [21], Bishoftu town [22], Gondar town [23], and Mekele town [14]. This may be due to government employed mothers couldn’t get enough time to frequently breastfeed their children.

Husband educational status was found a factor that affects the time to initiate CF. Similar finding was reported in Aksum [16], Harar [15] and Kenya [13]. This is due to the fact that education is believed to enable husbands to understand their wives and provide support to keep the child healthy, and this can help mothers to delay early weaning.

Mothers who did not have birth preparedness were at 3.74 times at risk of of introducing CF compared to their counter parts. Consistent finding was found in a study in Mekele town [14]. This can be due to the arrangement of conditions made before birth may help mothers to exclusively breastfeed their children and prevent early weaning.

Growth monitoring follow up was found a factor that affect the time to initiate CF. Supportive finding was found in Kamba district, southern Ethiopia [21]. This can be explained by mothers who get advice and health education during growth monitoring can exert a positive impact on delaying early weaning.

Mothers who did not know the exact time to introduce CF were 4.93 times at more hazard of introducing CF as compared to mothers who knew the exact time to introduce complementary feeding. Similar result was found in India [26], United Kingdom [5], West Africa [24], Bishoftu town [22], Jimma [25] Gondar town [23] and Kamba district [21]. This can be explained on the ground that the right knowledge can inspire mothers to start complementary feeding at the appropriate time.

Husband support during child feeding was found a factor that affect the time to initiate CF. this result is similar to those of studies conducted in Harar and Aksum towns [15, 16]. This can be due to mothers who get husband support during child feeding can get enough time to exclusively breastfeed their children and delay weaning.

### Conclusions

The median time to initiate CF was 6.00 months. The proportion of mothers who initiated into complementary feeding on time was 53.40%. Time to initiate into CF was affected by maternal employment, husbands educational status, birth preparedness, growth monitoring, ability to know the exact time to introduce complementary feeding, and husband support. Therefore establishing baby centres in government institutions, extending maternity leaves for reasonable months and focusing on parents with no formal education are important to improve timely initiation into CF.

### Limitations of the study

Due to retrospective nature of the study there might be a recall bias.

## Additional files

**Additional file 1.** Survival data set with minimal data.

## Abbreviations

AHR: adjusted hazard ratio
CF: complementary feeding
CHR: crude hazard ratio
COR: crude odds ratio
EDHS: Ethiopian Demographic and Health Survey
IYCF: infant and young child feeding
SVD: spontaneous vaginal delivery
VIF: variance inflation factor
WHO: World Health Organization

## References

[1] WHO, UNICEF. Strengthening action to improve feeding of infants and young children 6–23 months of age in nutrition and child health programmes. Report of proceedings Geneva. 2008.

[2] WHO, UNICEF. Global strategy for infant and young child feeding. 2003. 15806879

[3] Simondon KB, Simondon F (1997). Age at introduction of complementary food and physical growth from 2 to 9 months in rural Senegal. Eur J Clin Nutr.

[4] Popkin BM, Adair L, Akin JS, Black R, Briscoe J, Flieger W (1990). Breast-feeding and diarrheal morbidity. Am Acad Pediatr..

[5] Wright CM, Parkinson KN, Drewett RF (2004). Why are babies weaned early? Data from a prospective population based cohort study. Arch Dis Child.

[6] Gibson RS, Ferguson EL, Lehrfeld J (1998). Complementary foods for infant feeding in developing countries: their nutrient adequacy and improvement. Eur J Clin Nutr.

[7] Tang L, Lee AH, Binns CW (2015). Predictors of early introduction of complementary feeding: longitudinal study. Pediatr Int.

[8] Vaahtera M, Kulmala T, Hietanen A, Ndekha M, Cullinan T, Salin ML (2001). Breastfeeding and complementary feeding practices in rural Malawi. Acta paediatrica (Oslo, Norway: 1992).

[9] Kimani-Murage EW, Madise NJ, Fotso J-C, Kyobutungi C, Mutua MK, Gitau TM (2011). Patterns and determinants of breastfeeding and complementary feeding practices in urban informal settlements, Nairobi Kenya. BMC Public Health..

[10] UNICEF. Infant and young child feeding unite for children. 2011.

[11] Central Statistical Agency. Ethiopia mini demographic and health survey. 2014.

[12] Marriott BP, White A, Hadden L, Davies JC, Wallingford JC (2012). World Health Organization (WHO) infant and young child feeding indicators: associations with growth measures in 14 low-income countries. Maternal Child Nutr.

[13] CSA (2012). Ethiopia Demographic and Health Survey 2011 Addis Ababa Ethiopia and Calverton Mary land USA.

[14] Shumey A, Demissie M, Berhane Y (2013). Timely initiation of complementary feeding and associated factors among children aged 6 to 12 months in Northern Ethiopia: an institution-based cross-sectional study. BMC Public Health..

[15] Semahegn A, Tesfaye G, Bogale A (2014). Complementary feeding practice of mothers and associated factors in Hiwot Fana Specialized Hospital, Eastern Ethiopia. Pan Afr Med J.

[16] Yemane S, Awoke T, Gebreslassie M (2014). Timely initiation of complementary feeding practice andassociated factors among mothers of children aged from 6 to 24 months in Axum town, north Ethiopia. Int J Nutr Food Sci.

[17] Castro PD, Kearney J, Layte R (2015). A study of early complementary feeding determinants in the Republic of Ireland based on a cross-sectional analysis of the growing up in Ireland infant cohort. Public Health Nutr.

[18] Ogunlesi TA, Ayeni VA, Adekanmbi AF, Fetuga BM (2014). Determinants of timely initiation of complementary feeding among children aged 6–24 months in Sagamu, Nigeria. Nigerian J Clin Pract.

[19] Issaka AI, Agho KE, Page AN, Burns PL, Stevens GJ, Dibley MJ (2015). Factors associated with early introduction of formula and/or solid, semi-solid or soft foods in seven Francophone West African countries. Nutrients..

[20] Issaka AI, Agho KE, Page AN, Burns P, Stevens GJ, Dibley MJ (2014). Determinants of early introduction of solid, semi-solid or soft foods among infants aged 3–5 months in four Anglophone West African countries. Nutrients..

[21] Agedew E, Demissie M, Misker D, Haftu AD (2014). Early initiation of complementary feeding and associated factors among 6 months to 2 years young children, in Kamba Woreda, South West Ethiopia: a community-based cross-sectional study. Nutr Food Sci..

[22] Gemechu G, Adugna S, Damte M, Berhane Y (2015). Factors associated with early initiation of complementary feeding in Bishoftu Town, Oromia, Ethiopia. Open Access Lib J.

[23] Berhanu M, Zemene W, Mekonnen M (2015). Prevalence and associated factors of nonexclusive breastfeeding to infants within the first 6 months in Gondar Town, northwest Ethiopia, 2014.

[24] Issaka AI, Agho KE, Page AN, Burns PL, Stevens GJ, Dibley MJ (2015). Determinants of suboptimal complementary feeding practices among children aged 6–23 months in seven francophone West African countries. Maternal Child Nutr.

[25] Tamiru D, Aragu D, Belachew T (2013). Survey on the introduction of complementary foods to infants within the first six months and associated factors in rural communities of Jimma Arjo. Int J Nutr Food Sci..

[26] Patel A, Badhoniya N, Khadse S, Senarath U, Agho KE, Dibley MJ (2010). Infant and young child feeding indicators and determinants of poor feeding practices in India: secondary data analysis of National Family Health Survey 2005–06. Food Nutr Bull.

